# γ‐secretase inhibitor DAPT mitigates cisplatin‐induced acute kidney injury by suppressing Notch1 signaling

**DOI:** 10.1111/jcmm.13926

**Published:** 2018-11-08

**Authors:** Hitesh Soni, Anberitha T. Matthews, Sandeep Pallikkuth, Rajashekhar Gangaraju, Adebowale Adebiyi

**Affiliations:** ^1^ Department of Physiology University of Tennessee Health Science Center Memphis Tennessee; ^2^ Department of Ophthalmology University of Tennessee Health Science Center Memphis Tennessee; ^3^ Department of Anatomy and Neurobiology University of Tennessee Health Science Center Memphis Tennessee

**Keywords:** acute kidney injury, cisplatin, DAPT, Notch signaling

## Abstract

Organ toxicity, including kidney injury, limits the use of cisplatin for the treatment of multiple human cancers. Hence, interventions to alleviate cisplatin‐induced nephropathy are of benefit to cancer patients. Recent studies have demonstrated that pharmacological inhibition of the Notch signaling pathway enhances cisplatin efficacy against several cancer cells. However, whether augmentation of the anti‐cancer effect of cisplatin by Notch inhibition comes at the cost of increased kidney injury is unclear. We show here that treatment of mice with cisplatin resulted in a significant increase in Notch ligand Delta‐like 1 (Dll1) and Notch1 intracellular domain (N1ICD) protein expression levels in the kidneys. N‐[N‐(3,5‐difluorophenacetyl)‐L‐alanyl]‐S‐phenylglycine t‐butyl ester (DAPT), a *γ*‐secretase inhibitor reversed cisplatin‐induced increase in renal N1ICD expression and plasma or urinary levels of predictive biomarkers of acute kidney injury (AKI). DAPT also mitigated cisplatin‐induced tubular injury and reduction in glomerular filtration rate. Real‐time multiphoton microscopy revealed marked necrosis and peritubular vascular dysfunction in the kidneys of cisplatin‐treated mice which were abrogated by DAPT. Cisplatin‐induced Dll1/Notch1 signaling was recapitulated in a human proximal tubule epithelial cell line (HK‐2). siRNA‐mediated Dll1 knockdown and DAPT attenuated cisplatin‐induced Notch1 cleavage and cytotoxicity in HK‐2 cells. These data suggest that Dll1‐mediated Notch1 signaling contributes to cisplatin‐induced AKI. Hence, the Notch signaling pathway could be a potential therapeutic target to alleviate renal complications associated with cisplatin chemotherapy.

## 1 INTRODUCTION

Cisplatin, an anti‐tumor agent, is widely used as a part of treatment regimens for numerous human cancers, including mesothelioma, melanoma, neuroblastoma and esophageal, bladder, cervical, prostate, ovarian, testicular, lung, as well as head and neck cancers.^1^, ^2^, ^3^ Cisplatin kills cancer cells by crosslinking the purine bases on their DNA thereby disrupting replication and transcription.^2^ Although cisplatin as a primary cancer treatment or in combination with non‐platinum agents is highly effective, certain cancers are inherently resistant to the drug, while others acquire resistance after initial therapies.^2^, ^3^, ^4^, ^5^ The resistance of cancer cells to cisplatin chemotherapy is noteworthy due to its association with poor clinical outcomes including therapeutic failure and cancer recurrence.^2^, ^3^, ^5^

Apart from chemoresistance, cisplatin therapy is beset with marked adverse effects that hamper its clinical use. A rapid decline in kidney function (acute kidney injury) is a limiting factor in cisplatin chemotherapy and often results in treatment discontinuation.^6^, ^7^, ^8^ Up to 34% of cancer patients treated with cisplatin exhibited various degrees of nephrotoxicity.^9^, ^10^, ^11^ Hence, elucidation of the pathogenesis of cisplatin‐induced acute kidney injury (AKI) is essential for the development of adjunctive therapies to reduce morbidity and mortality in cancer patients.

The Notch signaling, a highly conserved cellular pathway controls cell fate specification, survival and differentiation.^12^, ^13^ The interaction between Notch ligands (Jagged1 and 2; Delta‐like1, 3 and 4) and their transmembrane receptors (Notch1‐4) on adjacent cells activate proteolytic cleavage of the Notch receptors by γ‐secretases thereby releasing the Notch intracellular domain (NICD) into the cytoplasm.^12^, ^13^ NICD translocates into the nucleus and forms a complex with transcriptional factors that regulate the expression of target genes.^12^, ^13^ Dysregulation of the Notch signaling pathway has been implicated in the pathophysiology of both cancer^12^, ^13^ and kidney disease.^14^, ^15^ Recent reports suggest that Notch signaling is upregulated in cisplatin‐resistant cancer.^16^, ^17^, ^18^, ^19^, ^20^ Accordingly, targeting the Notch signaling pathway with pharmacological inhibitors of γ‐secretase or knockdown of Notch components increased cisplatin efficacy against several cancer cells.^17^, ^21^, ^22^, ^23^, ^24^, ^25^, ^26^, ^27^ Whether enhancement of the anti‐cancer effect of cisplatin by Notch inhibition comes at the cost of increased kidney injury is unknown. In the present study, we investigated whether Notch signaling is induced in the kidneys of cisplatin‐treated mice. We also tested the hypothesis that pharmacological inhibition of the Notch signaling pathway ameliorates cisplatin‐induced AKI.

## 2 MATERIALS AND METHODS

### 2.1 Animals

All experimental animal procedures were reviewed and approved by the Animal Care and Use Committee of the University of Tennessee Health Science Center (UTHSC). Male mice (C57BL/6J; 8‐10 weeks old; Jackson Laboratories, Bar Harbor, ME, USA) were used in this study.

### 2.2 In Vivo studies

**Figure 1:**
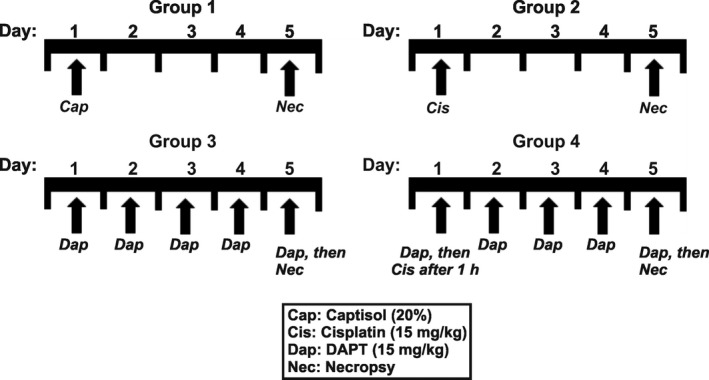
Schematic illustration of experimental groups. A group of mice was given a single IP injection of cisplatin (15 mg/kg). Mice in the control groups were treated (IP) with Captisol (vehicle) or DAPT (15 mg/kg) alone. Another group received DAPT 1 h before cisplatin administration, followed by daily DAPT injection for 4 days. The injection volume was kept at 10 μL/g body weight

Cisplatin and DAPT were solubilized in pharmaceutical excipient sulfobutyl ether‐β‐cyclodextrin (20% Captisol)^28^, ^29^, ^30^ as we have previously described.^31^ Mice were randomized into four groups (Figure 1; n = 16/group) and housed in ventilated micro‐isolation cages. A group of mice was given a single intraperitoneal (IP) injection of cisplatin (15 mg/kg). Mice in the control groups were treated (IP) with Captisol or DAPT (15 mg/kg) alone. Another group received DAPT 1 hour before cisplatin administration, followed by daily DAPT injection for 4 days. The dose of cisplatin used was chosen based on a previous study that determined the dose‐response nephrotoxic effect of cisplatin in mice upon single IP injections.^32^ The injection volume was kept at 10 μL/g body weight. On the fifth day, each mouse was weighed and placed on a new 96‐well plate inside an empty box for ~2 hours.^31^, ^33^ Urine samples were collected from the wells and analyzed. Blood was obtained from anesthetized mice via retro‐orbital bleeding. Kidneys were collected, weighed and processed after mice had been euthanized with sodium pentobarbital (200 mg/kg; IP) followed by exsanguination.

### 2.3 Determination of kidney function

To evaluate kidney function, we measured plasma or urinary concentrations of creatinine, urea nitrogen, cystatin C, neutrophil gelatinase‐associated lipocalin (NGAL) and albumin. We also determined the glomerular filtration rate (GFR) in the mice. Plasma and urinary creatinine concentrations were evaluated by mass spectrometry (isotope dilution LC‐MS/MS) at the O'Brien Core Center for Acute Kidney Injury Research (The University of Alabama at Birmingham, USA) as previously described.^34^, ^35^ Urine cystatin C and urine NGAL were determined with ELISA kits purchased from RayBiotech (Norcross, GA, USA). Blood urea nitrogen (BUN) and urine microalbumin levels were measured with kits purchased from Arbor Assays (Ann Arbor, MI, USA) and Exocell Inc. (Philadelphia, PA, USA), respectively. Active caspase 3 in kidney samples that were homogenized in a protease inhibitor‐free lysis buffer was determined with a Caspase 3 Colorimetric Assay Kit (BioVision, Inc., Milpitas, CA USA).

GFR was evaluated using the FIT‐GFR Inulin Kit and a one‐compartment plasma clearance method (BioPhysics Assay Laboratory; BioPAL, Worcester, MA, USA) according to the manufacturer's instructions and as previously described.^36^, ^37^, ^38^ Briefly, mice were injected with GFR‐grade inulin (5 mg/kg; IP). Blood samples were then collected at 30, 60 and 90 minutes post injection from the retro‐orbital plexus under isoflurane anesthesia. Inulin concentrations in plasma samples were measured using the BioPAL inulin ELISA plate. The data were fit to a one‐phase exponential decay equation *y = Be* ^*−bX*^, where *y* is inulin concentration, *B* is the intercept at time 0, *e* is the natural logarithm, *b* is the slope and *x* is the time. The GFR was calculated by dividing the dose of inulin given to the mice by the area under the curve (B/b) determined by integrating the exponential decay equation from time zero to infinity. The GFR was further normalized per gram kidney weight.

### 2.4 Histology

Kidney sections processed for Periodic acid–Schiff (PAS) staining were analyzed by a semi‐quantitative evaluation of tubular injury by renal pathologists at the Probetex Inc. (San Antonio, TX, USA) as we have previously described.^31^ Tubular injury scoring was based on the following scale: 0 = no apparent change; 1+ = focal: few focal areas distributed throughout the section; 2+ = infrequent: up to eight focal areas distributed throughout the section; 3+ = frequent: up to eight tubular profiles per 10× field; 4+ = very frequent: more than eight tubular profiles per 10× field.

### 2.5 Multiphoton intravital microscopy

An isoflurane‐anesthetized mouse was instrumented with a jugular vein catheter for the administration of fluorescence dyes, and the left kidney exteriorized via a small flank incision. To image the kidney, a mouse was placed on a heated (37°C) XYZ side stage (LSM Tech, Etters, PA, USA). The exteriorized kidney was rested in a glass bottom dish containing normal saline. Intravital imaging was performed using a Zeiss Axio‐Examiner Z1 two‐photon workstation (Carl Zeiss Inc., Thornwood, NY, USA) with a digitally controlled laser (Chameleon; Coherent Inc., Santa Clara, CA, USA). An objective inverter (LSM Tech) was used to convert the upright microscope into an inverted for side stage imaging. The mouse was injected with Hoechst 33342 (1 mg/kg), FITC‐dextran (200 kDa; 10 mg/kg), and propidium iodide (50 μg/kg) in 0.5 mL saline. The kidney cortex was viewed using a C‐Apo 40x water immersion objective (Carl Zeiss Inc.). Following animal imaging field stability, time‐series images in red, green and blue channels were collected. To examine blood flow in the peritubular capillaries, centerline scans of the vessels were performed after intravenous injection of FITC‐dextran.^39^, ^40^ The images were analyzed using the ImageJ software (NIH, Bethesda, MD, USA). Background‐subtracted fluorescence intensity of necrosis marker propidium iodide from randomly‐selected image fields was normalized to that of nuclear marker Hoechst 33342.

### 2.6 Quantitative RT‐PCR (qRT‐PCR)

Total RNA was isolated from snap‐frozen kidney samples using NucleoSpin^®^ RNA Plus kit (Macherey‐Nagel GmbH; Takara Bio USA). RNA samples (50 ng) served as a template for real‐time qRT‐PCR using EXPRESS SYBR Green reagents (Life Technologies, Carlsbad, CA, USA). The gene‐specific primers for Bcl‐2 were GAGTTCGGTGGGGTCATGTG (Sense), TAGTTCCACAAAGGCATCCCAG (Anti‐sense); Bax were CTGGATCCAAGACCAGGGTG (Sense), GTGAGGACTCCAGCCACAAA (Anti‐sense); 18S ribosomal RNA (18S rRNA) were CGAAAGCATTTGCCAAGAAT (sense), AGTCGGCATCGTTTATGGTC (Anti‐sense). The StepOnePlus Real‐Time PCR System (Applied Biosystems, Foster City, CA, USA) was used for PCR amplification. The expression levels of gene transcripts were determined using 2^−▵▵Ct^ and normalized to 18S rRNA.

### 2.7 Western immunoblotting

Kidney tissue and HK‐2 cell samples were homogenized in ice‐cold RIPA buffer. Proteins were separated by SDS‐polyacrylamide gel (4%‐20%) electrophoresis as we have previously described.^41^ Immunoreactive proteins were visualized and documented using a gel documentation system (Bio‐Rad, Hercules, CA, USA).

### 2.8 Human proximal tubule epithelial cell line (HK‐2)

The use of HK‐2 cell line was approved, and experiments were performed in accordance with the guidelines and regulations of the Institutional Biosafety Committee of the UTHSC. The cell line (CRL‐2190) was purchased from the American Type Culture Collection (Manassas, VA, USA). The cells were cultured as we have previously described.^31^

### 2.9 Apoptosis, cytotoxicity and cleaved Notch1 assays

Apoptosis of HK‐2 cells was examined in real time using the CellPlayer caspase‐3/7 reagent, and the IncuCyte ZOOM live content microscopy system (Essen BioScience, Ann Arbor, MI, USA) as we have previously described.^31^, ^42^ Cytotoxicity was also determined using the LDH colorimetric assay kit (Life Technologies). LDH release and percent cytotoxicity were quantified according to the manufacturer's instructions. The levels of cleaved Notch1 in HK‐2 cell lysates were evaluated with the PathScan Cleaved (Val1744) Notch1 ELISA kit (Cell Signaling Technology, Danvers, MA, USA).

### 2.10 siRNA transfection

Complexes consisting of a non‐targeting control or a pool of 3 target‐specific Dll1 siRNAs (Santa Cruz Biotechnology, Santa Cruz, CA, USA) and TransIT‐TKO Transfection Reagent (Mirus Bio, Madison, WI, USA) were prepared in Opti‐MEM medium (Life Technologies). HK‐2 cells were transfected with the siRNAs and maintained at 37°C; 5% CO_2_ for 72 hours. Western blotting was used to confirm effective knockdown of Dll1.

### 2.11 Antibodies and chemicals

Rabbit monoclonal anti‐Notch1 (intracellular; MilliporeSigma, Burlington, MA, USA; catalog #: 04‐1046), goat polyclonal anti‐Dll1 (Life Technologies; catalog #: PA519106), rabbit polyclonal anti‐Jag1 (mouse kidneys: Abcam, Cambridge, MA, USA; catalog #: ab7771), rabbit monoclonal anti‐Jag1 (HK‐2 cells: Life Technologies; catalog #: MA515012) and mouse monoclonal anti‐β‐actin (Abgent, Inc., San Diego, CA, USA; catalog #: AM1021). Unless otherwise specified, all chemicals were purchased from MilliporeSigma. Propidium iodide, DAPT and Captisol were purchased from Life Technologies, Selleck Chemicals (Houston, TX, USA) and CyDex Pharmaceuticals (Lenexa, KS, USA), respectively.

### 2.12 Data analysis

Statistical analysis was performed using the GraphPad Prism and InStat statistics software (Graph Pad, Sacramento, CA, USA). Statistical significance was determined with Student's t‐tests for paired or unpaired data and analysis of variance with Student‐Newman‐Keuls test for multiple comparisons. All data are expressed as mean ± standard error of the mean (SEM). A *P *< 0.05 was considered significant.

## 3 RESULTS

### 3.1 Cisplatin induces Notch signaling in mouse kidneys

**Figure 2:**
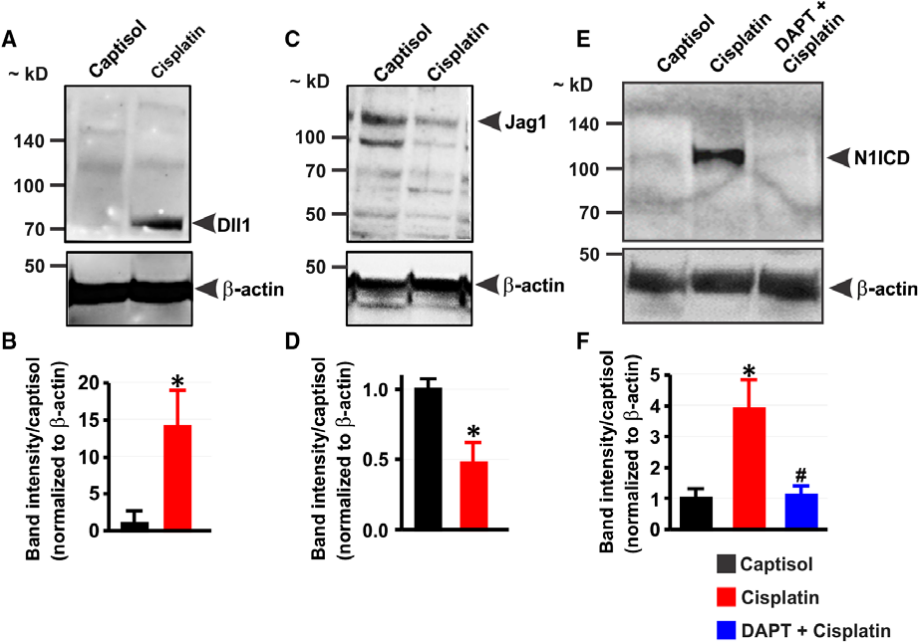
Cisplatin induces Notch signaling in mouse kidneys. A‐D, Western blot images and bar graphs showing Dll1 and Jag1 protein expression levels in the kidneys of mice 5 days after treatment with Captisol (vehicle control; IP) and cisplatin (15 mg/kg; IP). E and F, Western blot images and bar graphs illustrating N1ICD protein expression levels in the kidneys of Captisol‐, cisplatin‐, and DAPT (15 mg/kg; IP) + cisplatin‐treated mice. *P < 0.05 vs Captisol; ^#^ *P* < 0.05 vs cisplatin; n = 4 each

Two mice (1 each from Group 1 and Group 2) died before the completion of the study. The expression of Dll1 protein was almost undetectable in the kidneys of untreated mice but was induced in cisplatin‐treated mice (Figure 2A,B). By contrast, Jag1 expression was reduced ~ 2‐fold in the kidneys of cisplatin‐treated mice (Figure 2C,D). Furthermore, mice treated with cisplatin exhibited ~ 4‐fold increase in renal N1ICD protein expression when compared with the control (Figure 2E,F). Treatment of mice with DAPT abrogated cisplatin‐induced increase in renal N1ICD protein expression (Figure 2E,F). These data suggest that cisplatin stimulates Notch1 signaling in the kidney.

### 3.2 DAPT ameliorates cisplatin‐induced AKI in mice

**Figure 3:**
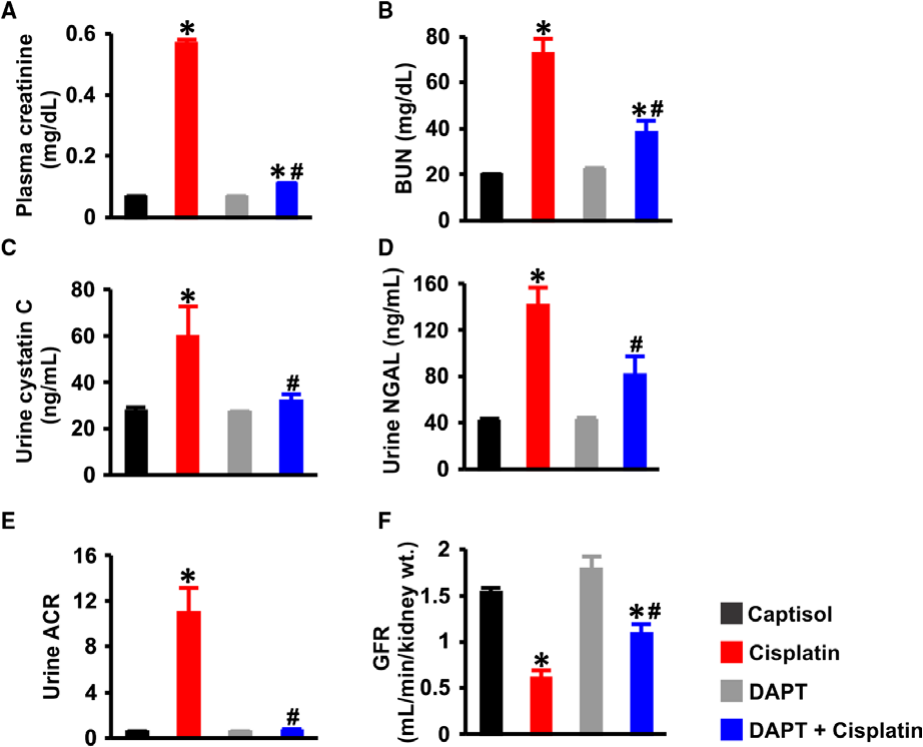
DAPT ameliorates cisplatin‐induced renal insufficiency in mice. Bar graphs summarizing: A, plasma creatinine (n = 6); B, BUN (n = 6); C, urinary cystatin C (n = 6); D, urinary NGAL (n = 6); E, urinary albumin‐creatinine‐ratio (ACR; n = 6); and F, GFR (n = 5 for all, except DAPT = 4) in Captisol (vehicle control; IP)‐, cisplatin (15 mg/kg; IP)‐, DAPT (15 mg/kg; IP)‐, and DAPT + cisplatin‐treated mice. *P < 0.05 vs Captisol; ^#^ *P* < 0.05 vs cisplatin

To test the hypothesis that Notch signaling contributes to cisplatin‐induced AKI, we measured the plasma or urinary levels of AKI biomarkers in the mice***.*** Plasma creatinine, plasma BUN, urine cystatin C, urine NGAL and urine albumin‐creatinine‐ratio (ACR) levels were increased ~ 8‐, 4‐, 2‐, 3‐ and 23‐fold, respectively in cisplatin‐treated mice (Figure 3A‐E). The plasma or urine concentrations of creatinine, BUN, cystatin C, NGAL and ACR in mice treated with DAPT alone were unchanged compared with the Captisol‐treated control (Figure 3A‐E). Moreover, DAPT ameliorated cisplatin‐induced increase in the levels of all the measured biomarkers (Figure 3A‐E).

The mean GFR in captisol‐treated control mice was ~ 1.5 mL/min/g kidney weight which was similar to that in mice treated with DAPT alone (Figure 3F). Cisplatin reduced GFR in the mice by ~ 51% (Figure 3F); an effect attenuated by DAPT (Figure 3F). These findings suggest (a) DAPT alone does not alter renal function, (b) Notch signaling induction contributes to AKI elicited by cisplatin and (c) DAPT alleviates cisplatin‐induced renal insufficiency in mice.

### 3.3 DAPT protects against renal morphological changes induced by cisplatin

**Figure 4:**
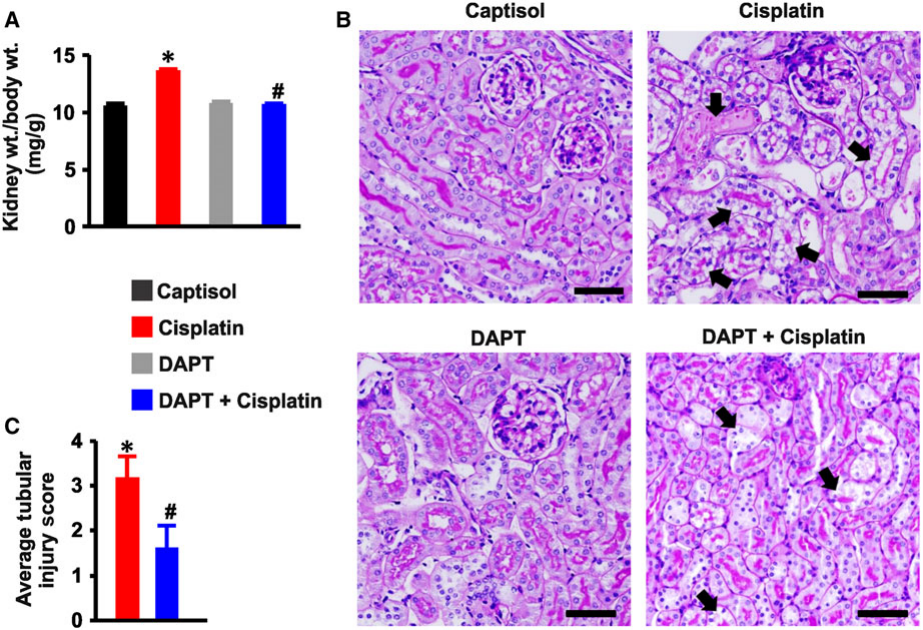
DAPT protects against renal morphological changes induced by cisplatin. A, kidney‐to‐terminal body weight ratio (n = 11 each; data were obtained from all groups, except mice used for multiphoton microscopy); B, images (PAS staining) and C, average tubular injury score (n = 4 each) in Captisol (vehicle control; IP)‐, cisplatin (15 mg/kg; IP)‐, DAPT (15 mg/kg; IP)‐, and DAPT + cisplatin‐treated mice. Average tubular injury scores in Captisol‐ and DAPT‐treated mice were zero. Arrows identify tubular injury in the form of vacuolar degeneration and necrosis. *P < 0.05 vs Captisol; ^#^ *P* < 0.05 vs cisplatin. Scale bar = 50 μm

The kidney‐to‐body weight ratio in Captisol‐ and DAPT‐treated mice was similar (Figure 4A). Cisplatin significantly increased the kidney‐to‐body weight ratio in the mice; an effect abrogated by DAPT (Figure 4A). There were no noticeable histopathological changes in the group of mice treated with the vehicle (Captisol) and DAPT alone, where tubules and glomeruli were normal in appearance (Figure 4B). Cisplatin caused tubular injury exemplified by overt necrosis and vacuolar degeneration which were attenuated by DAPT (Figure 4B,C). These data indicate that DAPT preserves renal morphology in cisplatin‐induced AKI.

### 3.4 DAPT mitigates necrosis, peritubular vascular dysfunction and apoptosis in the kidneys of cisplatin‐treated mice

**Figure 5:**
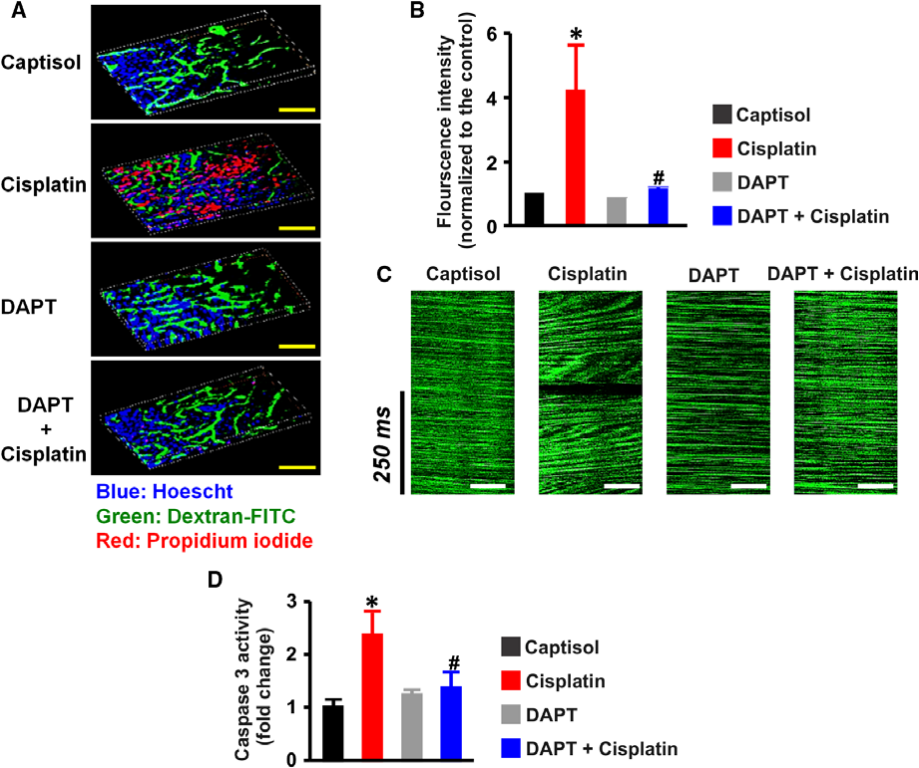
DAPT mitigates cisplatin‐induced necrosis, peritubular vascular dysfunction, and caspase 3 activity in mouse kidneys. A, 3‐D reconstruction of Z‐stack images and B, bar graphs of mean propidium iodide (PI) fluorescence intensity from 2‐photon microscopy of the kidney in anesthetized mice injected with Hoescht, dextran‐FITC, and propidium iodide to label the nuclei, peritubular vessels, and necrotic tubular cells, respectively in Captisol (vehicle control; IP; n = 4)‐, cisplatin (15 mg/kg; IP; n = 5)‐, DAPT (15 mg/kg; IP; n = 5)‐, and DAPT + cisplatin (n = 4)‐treated mice. C, representative line scan images of peritubular capillaries illustrating normal red blood cell velocity (characterized by constant slope) in Captisol‐, DAPT‐, and DAPT + cisplatin‐treated mice, and distorted perfusion (exemplified by variable slope) in cisplatin‐treated mice. D, bar graphs summarizing caspase 3 activity (determined by a colorimetric assay) in the kidneys of Captisol (n = 5)‐, cisplatin (n = 6)‐, DAPT (n = 6)‐, and DAPT + cisplatin (n = 6)‐treated mice; *P < 0.05 vs Captisol; ^#^ *P* < 0.05 vs cisplatin. Scale bar = 500 μm (yellow) and 50 μm (white)

Propidium iodide (PI), a cell membrane‐impermeant dye, labels necrotic cells with compromised membranes. Figure 5A panels show the 3‐D reconstruction of Z‐stack images of the renal cortex in anesthetized mice injected with nuclear stain Hoechst, dextran‐FITC and PI. Unlike Captisol‐ and DAPT‐treated mice, PI fluorescence was robustly induced in cisplatin‐treated mice (Figure 5A,B). Cisplatin‐induced increase in PI fluorescence intensity was reversed by DAPT (Figure 5A,B). Similarly, fast line‐scanning of peritubular capillaries showed distorted blood flow indicated by the variable slope in cisplatin‐treated mice compared with the constant slope in Captisol‐, DAPT‐ and DAPT + cisplatin‐treated mice (Figure 5C).

**Figure 6:**
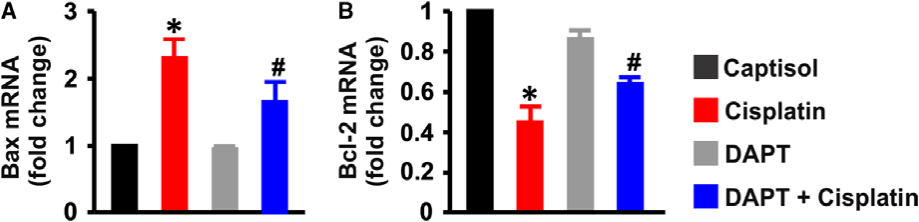
DAPT attenuates cisplatin‐induced Bax upregulation and Bcl‐2 downregulation in mouse kidneys. Bar graphs illustrating mRNA expression levels (n = 4 each) of A, Bax and B, Bcl‐2 genes in the kidneys of Captisol (vehicle control; IP)‐, cisplatin (15 mg/kg; IP)‐, DAPT (15 mg/kg; IP)‐, and DAPT + cisplatin‐treated mice; *P < 0.05 vs Captisol; ^#^ *P* < 0.05 vs cisplatin

Caspase 3 activity was elevated ~ 2‐fold in the whole kidneys of cisplatin‐treated mice compared with the mice treated with Captisol and DAPT (Figure 5D). Cisplatin‐induced caspase 3 activity was inhibited by DAPT (Figure 5D). Also, DAPT ameliorated cisplatin‐induced upregulation of pro‐apoptotic Bax and downregulation of anti‐apoptotic Bcl‐2 genes in the kidneys (Figure 6A,B). Together, these findings signify that cisplatin‐driven Notch signaling elicits renal cell death and peritubular vascular dysfunction.

### 3.5 Cisplatin‐induced Notch signaling promotes human proximal tubule epithelial cell death

**Figure 7:**
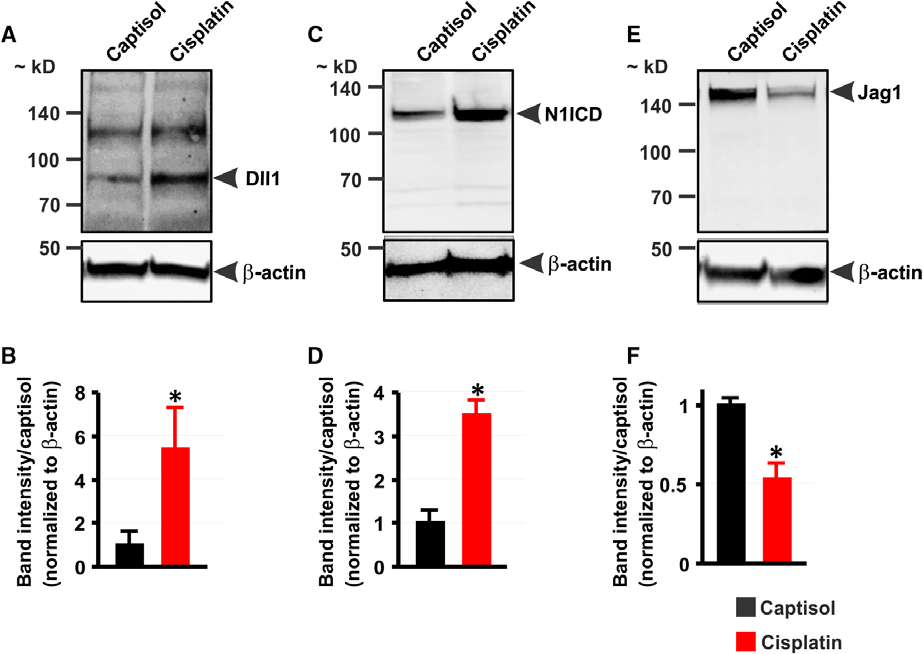
Cisplatin increases Dll1 and N1ICD but decreases Jag1 protein expression in HK‐2 cells. A‐F: Western blot images and bar graphs showing A and B, Dll1; C and D, N1ICD; and E and F, Jag1 protein expression levels in HK‐2 cells treated (12 h) with Captisol (vehicle control) and cisplatin (30 μmol L^−1^). *P < 0.05 vs Captisol; n = 3 each

**Figure 8:**
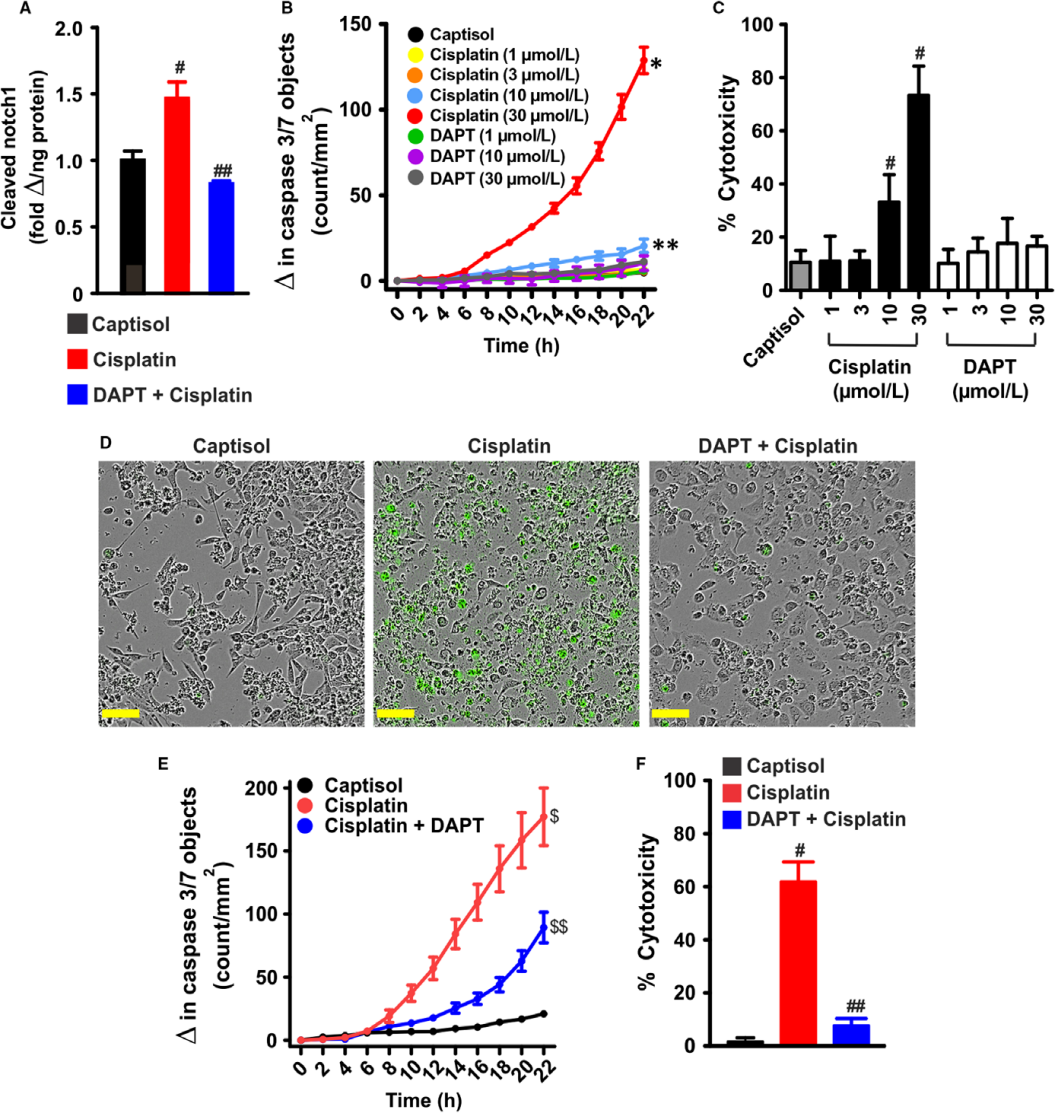
Cisplatin‐induced Notch signaling promotes human proximal tubule epithelial cell death. A, bar graphs summarizing the levels of cleaved Notch1 in Captisol (vehicle control)‐, cisplatin (30 μmol L^−1^)‐, and DAPT (10 μmol L^−1^) + cisplatin‐treated (12 h) HK‐2 cells (n = 6 each). B, kinetic curves (n = 4 each) demonstrating concentration‐ and time‐dependent effect of cisplatin and DAPT on caspase‐3/7 activity in HK‐2 cells. C, bar graphs (n = 5 each) showing percent cytotoxicity (LDH release) in Captisol‐, cisplatin‐, and DAPT‐treated HK‐2 cells. D, live content cell images (phase contrast and green fluorescent staining of nuclear DNA in apoptotic cells) and E, kinetic curves (n = 5 each) demonstrating that cisplatin (30 μmol L^−1^) induces time‐dependent increase in caspase‐3/7 activity in HK‐2 cells; an effect attenuated by DAPT (10 μmol L^−1^). F, bar graphs (n = 5 each) summarizing percent cytotoxicity in Captisol‐, cisplatin (30 μmol L^−1^)‐, and DAPT (10 μmol L^−1^) + cisplatin‐treated HK‐2 cells. ^#^ *P* < 0.05 vs Captisol; ^##^ *P* < 0.05 vs cisplatin; *P < 0.05 vs Captisol (8‐22 h); **P < 0.05 vs Captisol (14‐22 h); ^$^ *P* < 0.05 vs Captisol (12‐22 h); ^$$^ *P* < 0.05 vs cisplatin (12‐22 h). Scale bar = 300 μm

Next, we examined whether cisplatin‐induced Notch signaling in whole mouse kidneys will be recapitulated in cultured proximal tubule epithelial cells. Like in the kidneys of cisplatin‐treated mice, cisplatin increased Dll1 and N1ICD, but reduced Jag1 protein expression in HK‐2 cells (Figure 7A‐F). Cleaved Notch1 ELISA confirmed that cisplatin stimulated Notch1 cleavage; an effect abrogated by pretreating the cells with DAPT for ~ 30 minutes (Figure 8A). DAPT alone did not stimulate significant caspase 3/7 activation nor did it promote cytotoxicity in cultured HK‐2 cells (Figure 8B,C). By contrast, cisplatin concentration‐ and time‐dependently increased caspase‐3/7 activity and cytotoxicity in the cells (Figure 8B,C). Pretreatment of HK‐2 cells with DAPT attenuated cisplatin‐induced caspase 3/7 activity and cytotoxicity (Figure 8D‐F). Together, these data demonstrate that cisplatin triggers renal tubular Notch signaling, which may contribute to renal tubular cell death.

### 3.6 siRNA‐mediated Dll1 knockdown inhibits cisplatin‐induced Notch1 cleavage and apoptosis in HK‐2 cells

**Figure 9:**
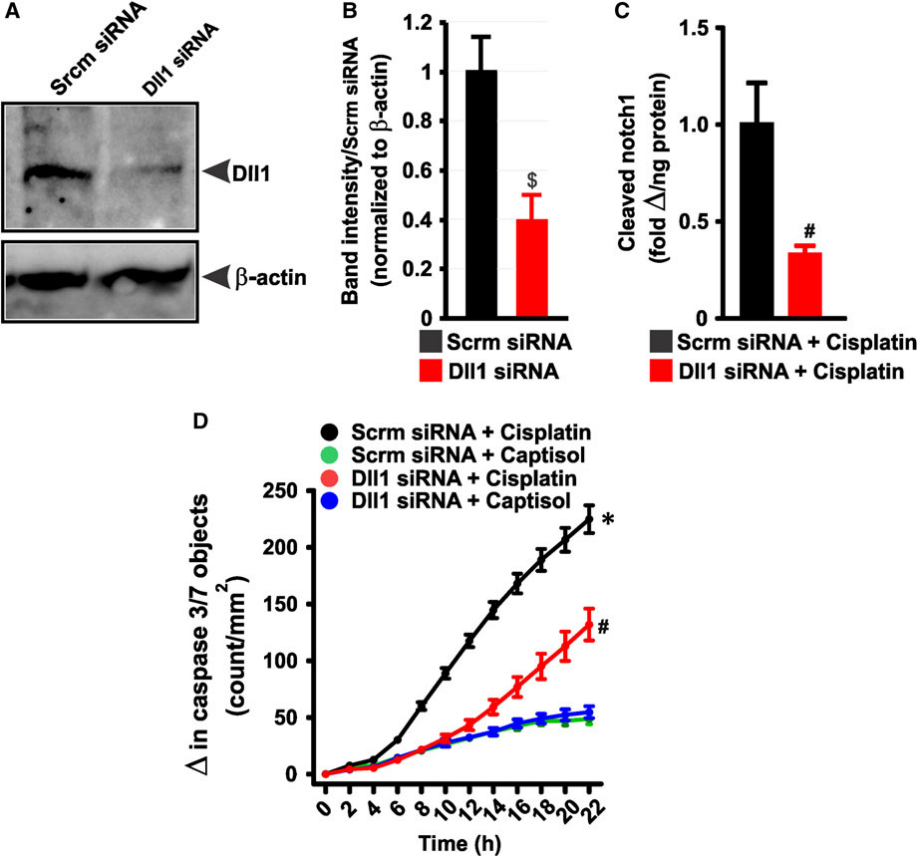
Dll1‐dependent Notch1 signaling contributes to cisplatin‐induced renal tubular cell death. A and B, Western blot images and bar graphs (n = 3) confirming Dll1 knockdown in Dll1 siRNA‐transfected HK‐2 cells. C, bar graphs summarizing the levels of cleaved Notch1 in scrambled (control; Scrm) siRNA‐ and Dll1 siRNA‐transfected HK‐2 cells (n = 6 each). D, kinetic curves (n = 12 each) demonstrating that cisplatin (30 μmol L^−1^)‐induced caspase‐3/7 activity in HK‐2 is diminished in Dll1 siRNA‐transfected HK‐2 cells. ^$^ *P* < 0.05 vs Scrm siRNA; ^#^ *P* < 0.05 vs Scrm siRNA + cisplatin (8‐22 h for “D”); *P < 0.05 vs Scrm and Dll1 siRNA + Captisol (8‐22 h)

To further investigate the hypothesis that Dll1‐mediated Notch1 signaling contributes to cisplatin‐induced renal tubular cell death, we examined cisplatin‐evoked Notch1 cleavage and caspase 3/7 activation in HK‐2 cells transfected with Dll1 siRNAs. Figure 9A,B confirmed Dll1 knockdown in siRNA‐transfected HK‐2 cells. Moreover, cisplatin‐induced Notch1 cleavage and caspase 3/7 activation were diminished in Dll1 siRNA‐treated cells compared with the control (Figure 9C,D). Our data indicate that Dll1‐dependent Notch1 signaling is involved in cisplatin‐induced renal tubular cell death.

## 4 DISCUSSION

Acute kidney injury is a common side effect of chemotherapeutic agent cisplatin.^7^, ^8^ Using in vivo and in vitro approaches, we demonstrated in this study that Notch signaling contributes to cisplatin‐induced AKI in mice. Cisplatin increased renal Notch ligand Dll1 but decreased Jag1 protein expression levels. Cisplatin also stimulated Notch1 cleavage and caused renal insufficiency and cell death which were reversed by DAPT, a γ‐secretase inhibitor. Moreover, Dll1 knockdown attenuated cisplatin‐induced Notch1 cleavage and apoptosis in human proximal tubule cells. Our findings provide new insights into the pathological mechanisms that underlie cisplatin‐induced nephrotoxicity.

The Notch pathway regulates nephrogenesis.^12^, ^43^ However, recent studies indicated that increased expression and activity of Notch components are associated with AKI and chronic kidney disease.^14^, ^15^ The expression levels of Notch ligands, receptors and transcriptional targets were all increased in rodents subjected to renal ischemia‐reperfusion.^44^, ^45^, ^46^ The Notch pathway is also induced in animals and human with diabetes, kidney fibrosis and glomerulosclerosis.^14^, ^15^ Accordingly, suppression of Notch signaling by inhibiting proteolytic cleavage of Notch receptors alleviated acute and chronic kidney disease.^14^, ^15^, ^44^, ^47^, ^48^ Data here signify that the basal protein expression level of N1ICD in mouse kidney is low. Additionally, we did not detect alterations in basal renal function in mice treated with DAPT alone. These findings corroborate previous reports suggesting that basal Notch signaling is reduced or absent in healthy adult kidneys.^14^, ^15^ Cisplatin increased Dll1 and N1ICD expression in mouse kidneys and proximal tubule epithelial cells. Inhibition of cisplatin‐induced Notch1 cleavage and renal cell death by DAPT and siRNA‐mediated Dll1 knockdown suggest that Dll1‐dependent Notch1 signaling contributes to cisplatin nephrotoxicity. It thus appears that a variety of renal insults may promote kidney damage by activating the Notch signaling pathway.

Our findings indicate that Dll1 stimulates N1ICD processing. Given that the Notch pathway is regulated by a variety of signaling molecules, including five ligands, our data did not unequivocally determine that Dll1 induction was solely responsible for N1ICD cleavage and renal injury in the mice. We show that cisplatin inversely altered dll1 and Jag1 expression in whole kidneys and cultured HK‐2 cells. It is conceivable that cisplatin triggers compensatory expression and function of renal Dll1 and Jag1 as Notch ligands may respond to different cellular signals or stimulate distinct Notch‐dependent biological processes.^49^, ^50^ Jag1 expression was increased in obstructed mouse kidneys and TGF‐β1‐treated renal cortical epithelial cells.^51^ Dll1, but not Jag1, Jag2, Dll3 or Dll4 expression levels were increased in the kidneys of rats subjected to ischemia/reperfusion.^46^ A study has also reported that cadmium chloride stimulated N1ICD cleavage and cell death while reducing Jag1 expression in HK‐2 cells.^52^ Together, these studies suggest that alterations in Notch ligand expression and activity in the kidney may depend on the type of renal insult.

Notch signaling is induced in hematologic malignancies such as T‐cell acute lymphoblastic leukemia and lymphoma, multiple myeloma and acute myelogenous leukemia as well as solid tumors.^53^, ^54^, ^55^ On the other hand, Notch signaling exhibited tumor‐suppressing activity in hepatocellular carcinoma, chronic myelomonocytic leukemia and head and neck squamous cell carcinoma.^53^, ^54^, ^55^ These reports indicate that the Notch signaling pathway elicits oncogenic or tumor suppressive activity, which may be due to variability in cancer cell‐type, tumor microenvironment, Notch dosage sensitivity and components of the Notch cascades that are activated.^53^, ^54^, ^55^ Notch signaling is upregulated in cisplatin‐resistant osteosarcoma, head and neck squamous cell carcinoma and lung, gastric and ovarian cancers.^16^, ^17^, ^18^, ^19^, ^20^ Suppression of Notch signaling by DAPT increased cisplatin cytotoxic efficacy against colorectal, nasopharyngeal, ovarian and lung cancer cells.^22^, ^25^, ^26^, ^27^ Therefore, DAPT and other inhibitors of the Notch signaling pathway may overcome cisplatin chemoresistance in responsive tumors.^56^, ^57^

Mechanisms of the protective effect of DAPT against cisplatin‐induced nephrotoxicity appears to include inhibition of caspase‐dependent and ‐independent cell death as cisplatin‐induced necrosis, caspase 3 activity, increase in pro‐apoptotic Bax and a compensatory decrease in anti‐apoptotic Bcl‐2 were all attenuated by DAPT. It is intriguing that DAPT inhibited cisplatin‐induced cell death in the kidney but promoted cisplatin‐induced cancer cytotoxicity.^22^, ^25^, ^26^, ^27^ Since pathological induction of the Notch signaling pathway can elicit cell growth or death,^12^, ^13^ cisplatin may differentially activate Notch‐dependent proliferation‐ and cell death‐associated genes in cancer and renal cells.

Exploration of combination therapy consisting of *γ*‐secretase inhibitors and cisplatin to circumvent chemoresistance is currently a subject of research interests.^56^, ^57^ Given the reversal of cisplatin‐induced nephropathy by DAPT, our study suggests that *γ*‐secretase inhibitors may kill the proverbial two birds with one stone by sensitizing responsive cancer cells to cisplatin and at the same time, protecting the kidneys against injury. Although the Notch signaling pathway is a major target of the proteolytic activities of *γ*‐secretases, other transmembrane proteins including the amyloid β precursor protein (AβPP) can be cleaved by the enzymes.^58^, ^59^ However, AβPP intracellular domain has been shown to degrade N1ICD, thereby inhibiting Notch1 signaling.^60^ Induction of both renal Dll1 and N1ICD by cisplatin and reversal of renal Notch1 cleavage by DAPT indicate that DAPT mitigates cisplatin‐induced AKI by suppressing Notch signaling.

In conclusion, our data suggest that Dll1‐mediated Notch1 signaling contributes to cisplatin‐induced AKI. Pharmacological inhibition of the Notch signaling pathway preserved renal function and morphology in cisplatin‐treated mice and viability in cisplatin‐treated human proximal tubule cell line. Hence, inhibition of Notch signaling could be a potential therapeutic strategy to alleviate renal complications associated with cisplatin chemotherapy.

## CONFLICT OF INTERESTS

The authors report no conflicts of interest.

## AUTHOR CONTRIBUTIONS

Study conception and design: A.A. Acquisition and analysis of data: H.S. performed animal and cell culture experiments; A.T.M. and A.A. performed cell culture and Western blot experiments; H.S., S.P. and A.A. performed 2‐photon microscopy experiments; R.G. performed qPCR experiments. Drafting of the manuscript: A.A. and R.G. Revision and approval of manuscript submission: All authors.
